# γ-Mangostin isolated from *Garcinia mangostana* L. suppresses inflammation and alleviates symptoms of osteoarthritis via modulating miR-124-3p/IL-6/NF-κB signaling

**DOI:** 10.18632/aging.103003

**Published:** 2020-04-16

**Authors:** Yen-Shuo Chiu, Jia-Lin Wu, Chi-Tai Yeh, Vijesh Kumar Yadav, Hsu-Shan Huang, Liang-Shun Wang

**Affiliations:** 1 Department of Orthopedics, Shuang Ho Hospital, Taipei Medical University, Taipei 23561, Taiwan; 2 School of Nutrition and Health Sciences, College of Nutrition, Taipei Medical University, Taipei 11031, Taiwan; 3 Research Center of Geriatric Nutrition, College of Nutrition, Taipei Medical University, Taipei 11031, Taiwan; 4 Department of Orthopedics and Traumatology, School of Medicine, College of Medicine, Taipei Medical University, Taipei 11031, Taiwan; 5 Department of Orthopedics, Taipei Medical University Hospital, Taipei 11031, Taiwan; 6 Department of Medical Research and Education, Taipei Medical University - Shuang Ho Hospital, New Taipei 23561, Taiwan; 7 Department of Health Care Management, Yuanpei University of Medical Technology, Hsinchu 30015, Taiwan; 8 The Division of Translational Medicine, Graduate Institute of Biomedical Informatics, Taipei Medical University, Taipei 11031, Taiwan; 9 Graduate Institute of Biomedical Informatics, College of Medical Science and Technology, Taipei Medical University, Taipei 11031, Taiwan; 10 Graduate Institute for Cancer Biology and Drug Discovery, College of Medical Science and Technology, Taipei Medical University, Taipei 11031, Taiwan; 11 Division of Thoracic Surgery, Department of Surgery, Shuang Ho Hospital, Taipei Medical University, New Taipei 23561, Taiwan

**Keywords:** OA, γ-MS, inflammation, miR-124-3p

## Abstract

Osteoarthritis (OA) a disease associated with joints and become severe with age, due to softening, inflammation and degradation of cartilage in joints. The agents that can target OA is needed, specifically without any side effects. *Garcinia mangostana* L. (Mangosteen) a tropical fruit used to treat many skin and stomach associated ailments. γ- Mangostin (γ-MS) a key bioactive substance present in mangosteen. Here, we aimed to explore γ-MS potential in targeting the pro-inflammatory cytokine, factors and miRs in OA progression. Significantly, γ-MS suppresses the inflammatory cytokines (IL-6, TNF-α, and INF- γ) and factors (NF-κB, STAT3, and COX-2) which regulates/participate in the catabolic process of cartilage destruction. Result of Hematoxylin-eosin (H&E) staining of tissue sections of OA joints of γ-MS treated and non-treated mice confirm γ-MS improves the signs of injuries, and maintains the structural integrity of the articular cartilage (epiphyseal disk joints and bone marrow) and reduces inflammation. Mechanistically, γ-MS targets miR-98-5p and miR-124-3p which are found to suppress the expression IL-6 and NF-κB, respectively. But in OA these miRs are inhibited, especially miR-124-3p which regulates not only NF-κB but also TNF-α, IL-6 and MMP7. With a further investigation underway, γ-MS represents an important source for treating and managing OA.

## INTRODUCTION

Osteoarthritis (OA) is a common form of the disease associated with joints in the body [1–4]. Mostly affecting the joints that have to bear the entire body weight, such as the knees, hips, hands, and spines [2, 3]. Common OA manifestations are associated with severe pain, stiffness, progressive destruction of cartilages and bones [5, 6]. But the cartilage degeneration mainly due to the biochemical and structural changes, or due to the imbalance between the catabolic and anabolic processes [7]. The etiology of OA includes various risk factors, such as joint injury, obesity, ageing, and heredity [8–11]. It has been estimated by 2020, OA will affect more than 50 million peoples in the United States. Studies suggest that the elevated level of pro-inflammatory factors, specifically interleukin (IL)-1β, IL-6, IL-8 and tumor necrosis factor-alpha (TNF-α), and these compounds may induce lipid mediators such as cyclooxygenase 2 (COX-2) enzymes were observed in both OA mouse models and patients [12–15]. These inflammatory factors may activate the nuclear factor-κB (NF-κB) signaling pathway to stimulates the catabolic process and leads to extracellular matrix (ECM) breakdown via up-regulated expression of metalloproteinase (MMP’s) [6, 12, 14]. Many cytokines levels are regulated by a DNA-binding molecule signal transducer and activator of transcription 3 (STAT3). Activation of STAT3 leads to increased pro-inflammatory cytokines and immune responses whereas the suppression of STAT3 improves OA [6, 16, 17]. The primary treatment of OA is mainly limited to analgesics, modified anti-rheumatic drugs, non-steroidal anti-inflammatory drugs, and glucocorticoids. However, these anti-inflammatory drugs have several adverse side effects and cannot completely alleviate the associated inflammation. Despite OA’s high prevalence, there are only fewer studies concerning the molecular mechanism and genetic factors in the pathogenesis of OA has been explored [18–20]. MicroRNAs (miRNAs) are large family of small 21-25 nucleotide non-coding RNAs. Recent evidence indicates that these small molecules regulate both the catabolism and anabolism of bone and cartilage [21, 22]. The importance of miRNA expression has been studied earlier to identify specific miRNAs that are differentially expressed in OA and other diseases [23–25]. miR expression is seen to affect the expression of pro-inflammatory cytokine and contribute to the pathogenesis of OA [26].

Developing a drug which is very safe and has no side-effects is challenging, because of that use of traditional and naturally available herbal medicines is getting more attention. *Garcinia mangostana* L. (Mangosteen) is a tropical fruit tree has been originally cultivated in Southeast Asia. It has long been consumed both as food and medicinal purposes, the dried pericarp of this fruits has been used particularly in treating ailments such as skin-related diseases, ulcers and diarrhea [27–29]. Mangosteen (MS) exudates a yellow resin is a rich source of xanthones and many bioactive substances, includes α-, β- and γ- Mangostin (γ-MS), garcinone B and E, along with mangostinone, tannins and flavonoids [30].

In this study, we aimed to examine the importance of mangosteen both *in vitro* and *in vivo*, Notably, γ-MS treatment suppresses the proinflammatory cytokines and factors, such as IL-6, NF-κB, STAT3, COX-2 TNF-α, and INF- γ in OA mouse model via up-regulating the miR-124-3p.

## RESULTS

### Enzyme induced OA

**Figure 1:**
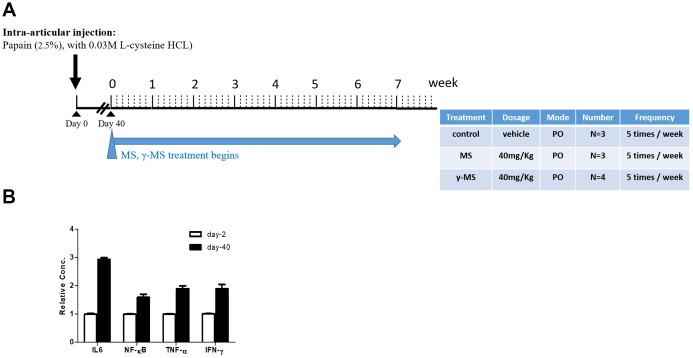
****Establishment of mouse OA model (**A**) Schematic representation of the timeline of chemically induced OA and mangostin treatment regimen. The table describes the treatment conditions. (**B**) Real-time PCR analysis comparing serum mRNA levels of IL-6, TNF-α, INF- γ and NF-κb before (healthy, day 0) and post OA induction (day 40). It was clear that after OA induction (day-40), the mRNA level of IL-6, NF-kB, TNF-α and INF-γ were significantly higher than un-induced counterparts (day-2).

We used papain-induced OA mouse model for evaluating the potential therapeutic effects of γ-mangostin. The overall experimental design was described in (Figure 1A and 1B). BALB/c mice were used for intra-articular injection of papain. After 40 days of papain treatment, serum mRNA levels of pro-inflammatory cytokines (IL-6, TNF-α and INF-γ) and the nuclear factor kappa-B (NF-κB) was found to be increased significantly in comparison to that of prior to OA induction. (Figure 1B). This finding served to mimic the biochemical and inflammatory responses during OA pathogenesis and the starting point for evaluating the effectiveness of our treatments with MS and γ-MS. mice.

### γ-MS inhibits inflammatory cytokines gene expression

**Figure 2:**
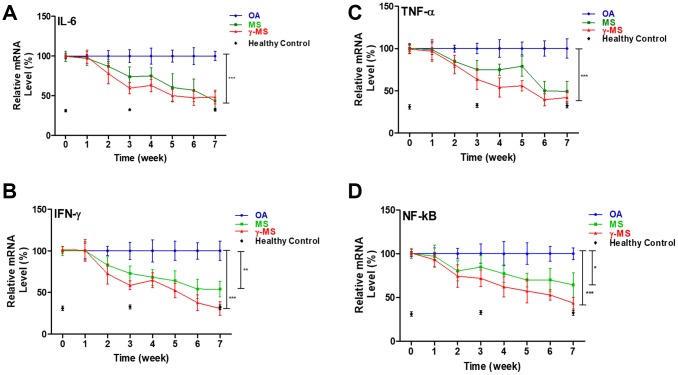
**Longitudinal qPCR analysis of serum inflammatory markers of OA.** (**A**–**D**) Relative ratio of mRNA expression of IL-6, TNF-a, IFN-γ and NF-κb between OA control; MS and γ-MS treated mice and healthy control. Three-time points (black diamonds) at week 0, 3 and 7 represent the relative mRNA level from the healthy control mice. *P<0.05; **P<0.01; ***P<0.005.

To evaluate the ability of MS and γ-MS in inducing the anabolic process of tissue repair, and reduces the activity of the inflammatory cytokine in OA. The inflammatory cytokines are the compounds contributing to the pathogenesis of OA [6]. Result of real-time polymerase chain reactions shows that treatment of MS and γ-MS significantly reduces the relative level of IL-6, TNF-α and INF-γ in comparison to control group (Figure 2A–2C). The expression level of NF-κB is also significantly reduced after the treatment (Figure 2D), NF-κB has been found to play an important role in the catabolic process and strongly associated with the development of OA [31].

### Therapeutic effect of γ-MS

**Figure 3:**
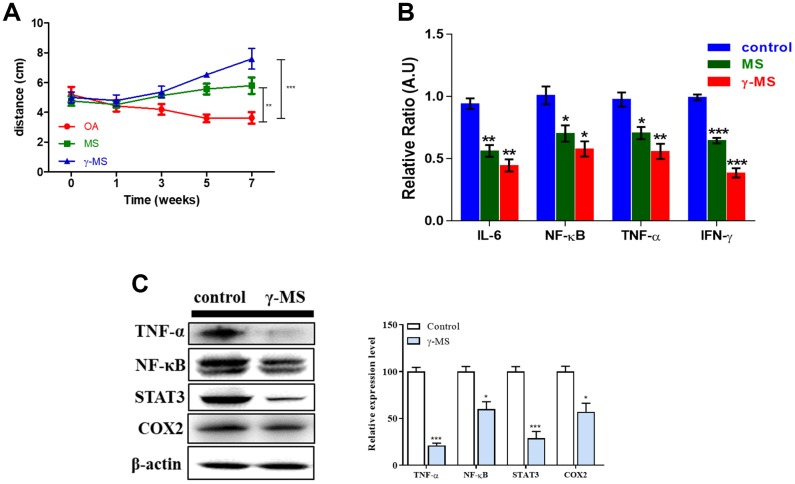
**γ-MS treatment improved the mobility in OA mice and downregulated the expression of inflammatory markers.** (**A**) Total distance travelled (during the test period) comparison of OA control; MS and γ-MS treated OA mice, significant recovery of OA is observed γ-MS treated mice in comparison to control and MS treatment group; (**B**) Significant decrease in expression of IL-6, TNF-α, INF-y and NFkB were observed in y-MS treatment group in comparison to the control group; (**C**) Western blot and bands quantified using densitometry analysis (ImageJ software). The treatment of γ-MS resulted in a markedly decrease in the expression of TNF-α, NFkB, STAT3, and COX2 as compared to the control group. The experiments were performed three times independently. Right panel represents the quantitative representation of 3 independent Western blot analyses. *P<0.05, **P<0.01 and ***P<0.001.

Although the mobility of OA patients appeared to be very limited and painful due to inflammation [32]. We confirmed that MS and γ-MS treatment improves the mobility significantly by subjecting treated and non-treated mice to an open field test to record their movement. Both the MS and γ-MS treated group mice moved more in comparison to the non-treated OA mice group (Figure 3A). Interestingly, γ-MS treated group shows better response (***p<0.001) and improvement in walking than MS (**p<0.01) and non-treated mice group. Over the period of treatment, the relative mRNA expression of IL-6, TNF-α, INF-γ and NF-κB reduced significantly in γ-MS treated group (Figure 3B). To evaluate the effect of γ-MS on human synovial fibroblast cell line SW982 for checking the change in expression OA associated factors. We further analyzed the protein expression level of TNF-α, NF-κB, STAT3 and COX2 by using western blotting. The results show γ-MS treatment reduces the expression of TNFα, NF-κB, STAT3 and COX-2 in comparison to non-treated samples (Figure 3C).

### γ-MS treatment-induced therapeutic effects were associated with an increased miR-124-3p level

**Figure 4:**
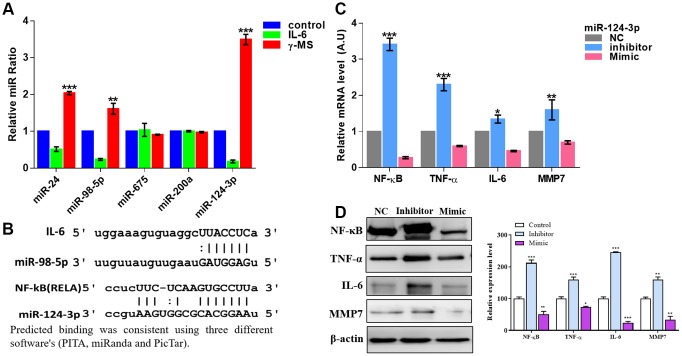
**γ-MS mediated effects were associated with increased miR-124-3p.** (**A**) qPCR analysis of different inflammation-associated miRs. SW982 cells were stimulated with IL-6 and showed a significantly decreased level of miR-24, miR-98-5p and miR-124-3p (the most responsive miR) while γ-MS treatment significantly increased these miRs. (**B**) Mir-Target prediction shows the 3’UTR sites of IL-6 and NF-κB respectively targeted by miR-98-5p and miR-124-3p. (**C**, **D**) qPCR and Western blot analysis (quantified band by densitometry analysis using ImageJ software) demonstrated that inhibitor of miR-124-3p led to an elevated level of inflammatory markers, TNF-α, IFN- γ, IL-6 and MMP7 while the mimic molecule reduced the level. *P<0.05, **P<0.01 and ***P<0.001.

A number of miRNAs have been studied earlier and found to be involved in the pathogenesis of OA [33, 34]. These miRNAs regulate the expression of both anabolic and catabolic associated genes, and the importance of epigenetic regulation of gene expression to the development of OA has been reported earlier [33, 34]. After stimulating the SW982 cells with IL-6, qRT-PCR analysis shows the significant decrease in the relative ratio of miR-24, miR-98-5p and miR-124-3p, whereas the most significant effect, is on miR-124-3p (Figure 4A), after γ-MS treatment this miRs significantly increased (Figure 4A). Predicted binding of these miRNA from online databases such as PITA [35], miRanda [36] and PicTar [37] shows IL-6 and NF-κB were targeted by miR-98-5p and miR-124-3p respectively (Figure 4B). Furthermore, a reporter assay for miR-124-3p was also demonstrated the direct binding of miR-124-3p with RELA (NF-kB) as described in Supplementary Figure 2. Inhibition of miR-124-3p led to an increase in the level of inflammatory markers, viz, NF-κB, TNF-α, IL-6 and MMP7 as observed both by qRT-PCR and western blotting (Figure 4C, 4D).

### Histopathological analyses in OA tissues after γ-MS treatment

**Figure 5:**
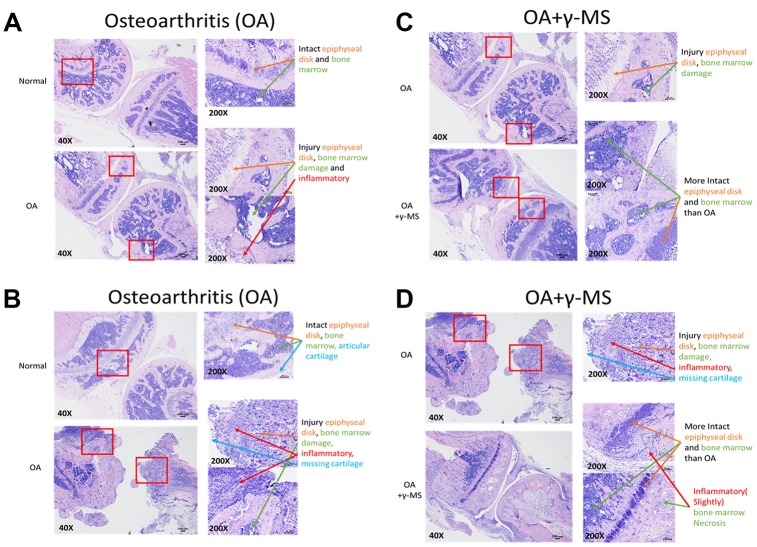
**Comparative immunohistochemical analysis between the knee joints from control and γ-MS treatment groups.** The representative section of OA mice demonstrated signs of cartilage degradation and tissue injuries (epiphyseal disk and bone marrow) and inflammation in comparison to healthy normal counterparts (**A**, **B**). γ-MS treated mice showed a lower degree of cartilage destruction and more intact epiphyseal disk and bone marrow. as well as less inflamed in comparison to non-treated OA control group (**C**, **D**). H & E- stained images were taken at 40X (Bar = 100μM) and 200X magnification (Bar = 50μM).

Next, we determined the efficacy of γ-MS on BALB/c OA mice through oral administration by evaluating the structural integrity of the articular cartilage injury. H & E staining of OA joint sections isolated from the mice revealed detectable degradation in the epiphyseal disk joints tissues, inflammation, degradation of cartilage and bone marrow necrosis in comparison to normal mice tissue (Figure 5A, 5B). γ-MS treated OA mice tissue sections shows signs of reparative resynthesis effect on epiphyseal disk joints tissues, intact bone marrow and inflammation is also reduced in comparison to non-treated OA control group mice (Figure 5C, 5D), these sections were further evaluated and graded by two independent experienced pathologists, the grade score also suggests signs of healing and repair of tissues because of γ-MS treatment (Table 1). Safety of γ-MS treatment on normal tissue was also evaluated, there was no drug side effect in normal mice. There is no difference between the γ-MS treated and non-treated group tissue epiphyseal disk and bone marrow were intact (Supplementary Figure 1).

**Table 1:** Grading for histopathological changes in OA tissues after γ-MS treatment.

**Groups**	**Observer 1**	**Observer 2**	Average grading (Score*)
Normal control	0	0	G 0
Osteoarthritis (OA)	3	3	Grade 3.0
OA + γ-MS	1.5	2	Grade 1.5

*The grading scores presented here represent the average score obtained from 2 separate pathologists.

**Table:** Histological grading scheme (McNulty et al., 2011)*

**Articular cartilage structure score (ACS)**	
**Grade (key feature)**	**Associated criteria (tissue reaction)**
Grade 0	Articular surface morphology is intact and smooth
Grade 1-3	Fibrillation/clefts and loss of cartilage involving one by fourth or less of articular cartilage thickness
Grade 4-6	Fibrillation and/or clefts and/or loss of cartilage involving one half or less of articular cartilage thickness
Grade 7-9	Fibrillation and/or clefts and/or loss of cartilage involving more than one half of articular cartilage but less than full thickness
Grade 10-12	Fibrillation and/or clefts and/or loss of cartilage involving the full thickness of articular cartilage

*McNulty MA, Loeser RF, Davey C, Callahan MF, Ferguson CM, Carlson CS. A Comprehensive Histological Assessment of Osteoarthritis Lesions in Mice. Cartilage. 2011; 2:354–63. https://doi.org/10.1177/1947603511402665 PMID:3741515

## DISCUSSION

In this study, we demonstrated the evidence both *in vivo* and *in vitro* that γ-MS administration suppressed the progression of OA in the chemically (papain) induced mouse model. Specifically, we showed that the γ-MS treatment delayed OA pathogenesis via suppressing chronic inflammation.

OA is characterized by both acute and chronic inflammations mostly in articular joints. In the late stage of OA, the destruction of the joints results in severe functional loss and disabilities in the patients. OA incidences have increased and become a major health issue for the ageing population [9, 38]. Complex pathogenic mechanisms are involved in OA. Particularly, the articular synovial tissues are thought to play an important role, as both intra-articular cartilage lesions and secondary inflammatory lesions caused by synovial inflammations. That can facilitate the occurrence and advancement of OA, further severing the clinical symptoms [39, 40]. In addition, fibrosis of synovial tissues triggers a large number of inflammatory cells and pro-inflammatory cytokines, and associated factors which lead to the pathogenesis and progression of OA [41]. In healthy cartilage proper balance of anabolic and catabolic activities are important for the maintenance of cartilage tissue integrity and the repair of molecular damages. When this balance is compromised it leads to OA condition [42]. Tissue homeostasis is controlled by coordinated crosstalk among the factors that regulate this balance. Pro-inflammatory cytokines such as IL-6, TNF-α, INF-γ involved in catabolic process in cartilage degradation and disease progression [43]. In addition, NF-κB signaling pathways have also been prominently demonstrated in the pathogenesis of OA [44]. NF-κB is activated by inflammatory cytokines and acts in a positive feedback loop to stimulate the catabolic process by stimulating the inflammatory cytokines (ADAMTs) and enzymes MMPs that further leads in destruction of cartilage [44]. The observations that γ-MS treatment significantly reduced the expression of aforementioned inflammatory markers strongly suggests its potential as a disease-modifying agent for OA Currently, non-steroidal anti-inflammatory drugs (NSAIDs) are used for pain management because of their ability to target COX-2, but it has unwanted gastrointestinal effects.

Administration of γ-MS to OA induced mice model showed a decrease in the level of expression of these pro-inflammatory cytokines and the transcriptional factor which further stimulates the pathogenicity and severity of OA (Figure 2A–2D). Also, the mobility of OA mice treated with γ-MS was measured (Figure 3A), and treatment group OA mice were able to walk further comparison to non-treated mice. Significant decrease in serum mRNA levels of inflammatory cytokines and transcription factors were also observed for treatment group both by qRT-PCR and western blotting (Figure 3B–3C).

Our result also shows γ-MS targets and reduces the expression of NF-κB and STAT3 both of which are associated with catabolism and imbalances the body homeostasis. Expression of COX-2 (Figure 3C) was also targeted by γ-MS which is a major therapeutic target for pain management in the clinical settings. According to our data, γ-MS appeared to suppress a broader spectrum of inflammatory markers as compared to COX-2 inhibitors.

miRs are now becoming a great tool, that could play a key role in OA by regulating hundreds of genes that were important in homeostasis (catabolic and anabolic process) and OA pathology [45]. Especially, studies explained that miRNA-29 family targets NF-κB [46], miRNA-25 targets COX-2 [46], miRNA-149 and miRNA-98 targets TNF-α and IL-6 [23, 46] and they play an important part in OA. The qRT-PCR analysis shows γ-MS treatment significantly increased the miRs that were inhibited in IL-6 treated SW982 cells. For example, miR-24, miR-98-5p and miR-124-3p. Especially the expression of miR-98-5p and miR-124-3p which are predicted to target IL-6 and NF-κB from prediction software (Figure 4A, 4B). Importantly inhibition of miR-124-3p resultant in the elevated level expression of inflammatory factors (NF-κB, TNF-α, IL-6 and MMP7) both at mRNA and protein level as compared to the control mimic treated group.

Intra-articular cartilage and inflammatory lesions caused by synovial inflammations can facilitate advancement and severing of OA [39, 40]. Our immunohistochemical analyses of the knee joint provided strong evidence that γ-MS treatment prevented the further destruction of the articular cartilage after injury or in case of OA. It was clear that γ-MS treated samples showed noticeable higher integrity of epiphyseal disk joints tissues, intact bone marrow and reduction in inflammation (Figure 5A–5D) in comparison to non-treated OA control group mice.

## CONCLUSIONS

This study provides evidence that γ-MS treatment both *in vitro* and *in vivo* significantly delayed pathogenesis of OA. γ-MS targets signaling molecules and regulators that contributes to inflammation and degradation of cartilages, which compromised the anabolic and catabolic balance of body homeostasis in OA. Hence, natural product (γ-MS) may function to manage OA and further development into the clinical settings is warranted.

## MATERIALS AND METHODS

### Preparation of mangostin and cell culture

Mangostin (MS) was purchased from Sigma Aldrich (catalogue ns. PHL89247purity is ≥98% (HPLC) while gamma (γ-MS) was provided by Genhealth Pharma, Taiwan Both MS and γ-MS were dissolved in dimethyl sulphoxide (DMSO) and stock of 100mmol/L prepared stored at -20°C. Stock solutions were diluted before the experiment each time. The human synovial sarcoma cells SW982 obtained from the American Type Culture Collection (ATCC) and were cultured according to ATCC’s recommended conditions. SW982 has been established as a common cell model as a surrogate for synovial fibroblasts for studying arthritis [47]. For IL-6 stimulation, SW982 cells were cultured with IL-6 (100ng/mL, 24h) to serve as an inflamed cell model, a protocol adapted from a previously established study [48]; subsequently, IL-6 induced SW982 cells were treated with γ-MS (5μM, 24h) and harvested for further analyses including qPCR and western blots.

### Animals

BALB/c mice, 6 weeks of age, were used as per the protocols approved by the Animal Use Protocol Taipei Medical University.

### Induction of osteoarthritis and drug administration

For the induction of OA in animals, 0.2ml of papain (P4762; SIGMA) and 0.1ml of cysteine were administered intra-articularly (2.5% papain and 0.03M L-cysteine HCL prepared in distilled water) accordingly [49, 50]. After the OA induction (please refer to Figure 1 for experimental design), the animals were divided into 4 groups, healthy controls (n=3), OA (vehicle control, n=3), MS (n=3), and γ-MS (n=4) treatment groups. MS and γ-MS were administrated orally (per os; p.o) at 40mg/kg dissolved in corn oil; doses five times a week.

### Hematoxylin-eosin (H&E) analysis

After 18 weeks, mice in the groups were humanely sacrificed by dislocating the neck, fresh knee cartilage tissues were collected and then preserved in 40g/L paraformaldehyde solution with phosphate buffer saline (PBS) for 22h. Fixed specimens were decalcified in 0.5 mol/L ethylenediaminetetraacetic acid (EDTA) solution for 30 days, and processed. Then a vertical incision was made to the articular cartilage with the scalpel blade. Specimens were then dried by automatic dehydrator and then embedded into paraffin, conventional histopathological slides were collected with 4μM sections. After dewaxing and dehydration of section, H&E staining was performed as previously described [51]. All the sections were carefully examined histologically, and the changes in cellular morphology were observed under a microscope at a magnification of X40 and X200.

### Real-time PCR

The total RNA was isolated and purified using TRIzol-based protocol (Life Technologies) according to the manufacturer’s instructions. Hundred nano-grams of total RNA was reverse transcribed using QIAGEN One-step RT-PCR Kit (QIAGEN, Taiwan), and the PCR reaction was performed using a Rotor-Gene SYBR Green PCR Kit (400, QIAGEN, Taiwan).

The primers sequence for IL-6 were F: 5'- TAGCCGCCCCACACAGACAG-3'; R: 5'- GGGTTGGTGTTTACGGTCGG-3'. The primer sequences for NF-κB were F: 5' GCGTACACATTCTGGGGAG -3'; R: 5'- CCGAAGCAGGAGCTATCAA -3'; the primer sequences for TNF-α were F: 5'- CCTGTAGCCCACGTCGTAGC-3'; R: 5'- AGCAATGACTCCAAAGTAGACC-3'; the primer sequences for IFN-γ were F: 5'- CTCTTGGCTGTTACTGCCAGG-3', R: 5'- CTCCACACTCTTTTGGATGCT-3'; the primer sequences for MMP-7 were F: 5'- TCCCGCGTCATAGAAATAATG-3', R: 5'- AGGAATGTCCCATACCCAAAG-3'. The primer sequences for the internal control RPLP0 were F: 5'-TGGTCATCCAGCAGGTGTTCGA-3', R: 5'-ACAGACACTGGCAACATTGCGG-3'.

The qRT-PCR for murine primer sequence given in Supplementary Table 1 and the microRNA primer that are used in this study were purchased from QIAGEN (QIAGEN, Taiwan), detailed catalogue number was enumerated in Supplementary Tables 1 and 2.

### Western blot

Total proteins of Human synovial sarcoma derived cell line (SW982) were extracted after treatment from different experiments were separated using the SDS-PAGE using Mini-Protean III system (Bio-Rad, Taiwan) and transferred onto PVDF membranes using Trans-Blot Turbo Transfer System (Bio-Rad, Taiwan). Membranes were incubated overnight at 4°C in following primary antibodies NF-κB (#6956S), TNF-α (#3707), IL-6 (#12912), MMP7 (Sc-8832), STAT3 (#4904), COX-2 (#4842) and β-actin (Sc-47778). Secondary antibodies were purchased from Santa Cruz Biotechnology (Santa Cruz, CA) and ECL detection kit was used for the detection of the protein of interests. Images were captured and analyzed using UVP BioDoc-It system (Upland, CA, USA).

### Statistical analysis

All experiments were performed in triplicate. The statistical significance was calculated by unpaired two-tailed Student’s t-test where more groups were involved, All the statistical analyses were performed using GraphPad Prism software where a p-value <0.05 was considered as statistically significant and was indicated with an asterisk.

## Supplementary Material

Supplementary Figures

Supplementary Tables
