# Supplementary material for: γ-Mangostin isolated from Garcinia mangostana L. suppresses inflammation and alleviates symptoms of osteoarthritis via modulating miR-124-3p/IL-6/NF-κB signaling
Source: Aging (Albany NY). 2020 Apr 16;12(8):6630–43. doi: 10.18632/aging.103003 (PMC7202528; doi:10.18632/aging.103003)
Supplement: Supplementary Figures [file aging-12-103003-s002..pdf]

SUPPLEMENTARY FIGURES

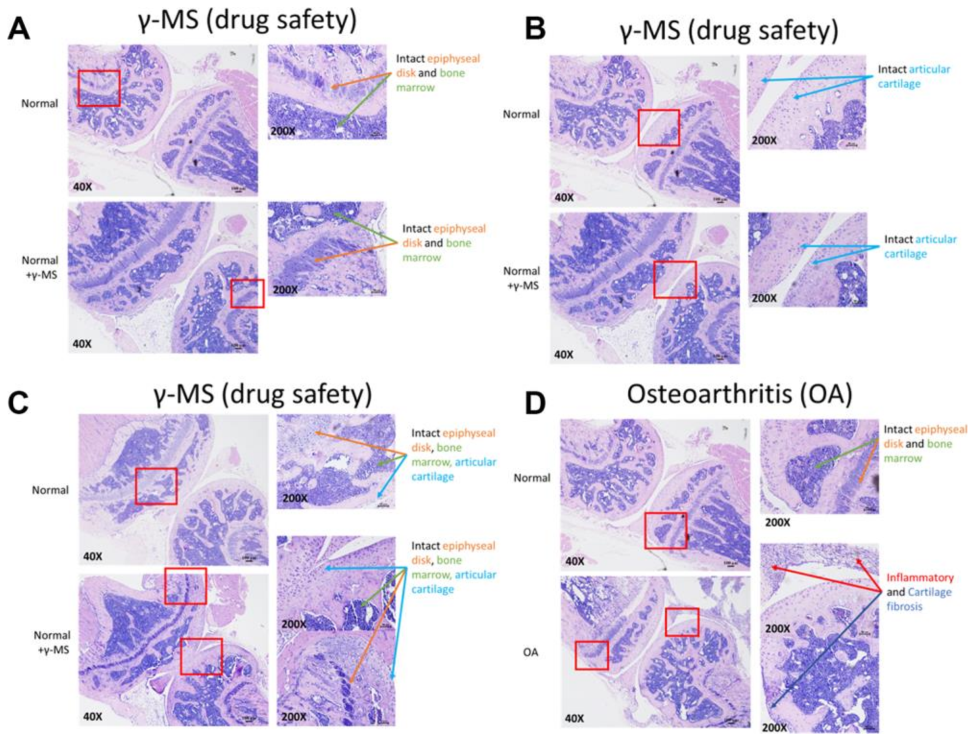

Supplementary Figure 1. H & E staining of normal knee control. (**A**–**D**) γ-MS treatment is also not showing any drug side effect in normal mice. There is no difference between the treated and non-treated group tissue (epiphyseal disk and bone marrow). H & E- stained images were taken at 40X (Bar = 100μM) and 200X magnification (Bar = 50μM).

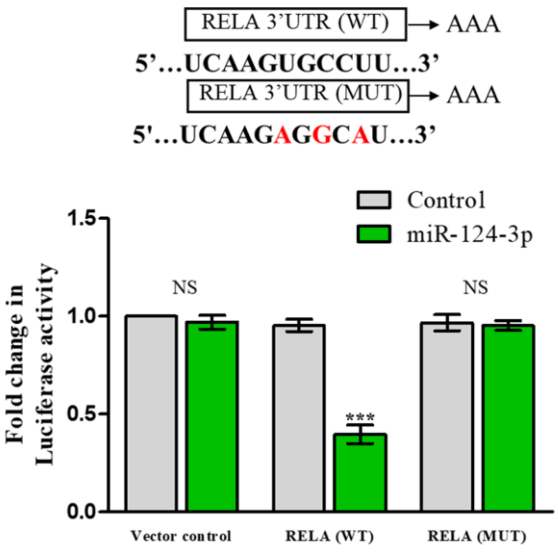

Suppleemntary Figure 2. Luciferase reporter assay. The insert demonstrates the sequences of 3'UTR of RELA (WT, wild type) and mutant (MUT, red letters, altered nucleotides) used for demonstrating the direct binding of miR-124-3p with RELA (NF-kB). The luciferase activity was significantly lower when SW982 cells were transfected with RELA (WT) and miR-124-3p, while no difference was observed in both vector control and RELA (MUT). ***P<0.001; NS, not significant
