# Supplementary material for: γ-Mangostin isolated from Garcinia mangostana L. suppresses inflammation and alleviates symptoms of osteoarthritis via modulating miR-124-3p/IL-6/NF-κB signaling
Source: Aging (Albany NY). 2020 Apr 16;12(8):6630–43. doi: 10.18632/aging.103003 (PMC7202528; doi:10.18632/aging.103003)
Supplement: Supplementary Tables [file aging-12-103003-s001..pdf]

SUPPLEMENTARY TABLES

Supplementary Table 1. Murine primer sequence.

miRNA	Murine Primer Sequences
Mouse TNF-α -F	TCTCATGCACCACCATCAAGGACT
Mouse TNF-α -R	TGACCACTCTCCCTTTGCAGAACT
Mouse NF-κB -F	GGGCAGTGACGCGACG
Mouse NF-κB -R	AGCGCCCCTCGCATTTATAG
Mouse IFN-γ -F	CGGCACAGTCATTGAAAGCCTA
Mouse IFN-γ -R	GTTGCTGATGGCCTGATTGTC
Mouse RPLP0 -F	AGATTCGGGATATGCTGTTGGC
Mouse RPLP0 -R	TCGGGTCCTAGACCAGTGTTC
Mouse IL-6 -F	GAGGATACCACTCCCAACAGACC
Mouse IL-6 -R	AAGTGCATCATCGTTGTTCATACA

Supplementary Table 2. miRNA primer details.

miRNA	QIAGEN catalogue number
hsa-miR-24	MS00006552
hsa-miR-98-5p	MS00003367
hsa-miR-675	MS00032109
hsa-miR-200a-3p	MS00003738
hsa-miR-124-3p	MS00006622
